# Effects of photo-assisted electrodeposited on CuInSe_2_ thin films

**DOI:** 10.1186/1556-276X-9-660

**Published:** 2014-12-09

**Authors:** Tsung-Wei Chang, Wen-Hsi Lee, Yin-Hsien Su, Yu-Jen Hsiao

**Affiliations:** 1 National Cheng Kung University, No.1, University Road, Tainan City 701, Taiwan; 2 National Nano Device Laboratories, No.1, University Road, Tainan City 701, Taiwan

**Keywords:** CIS, Solar cells, Electrodeposition, Photo-assisted

## Abstract

Photo-assisted one-step electrodeposition has been applied to help in forming smooth and dense CuInSe_2_ films. The difference in surface morphology and crystalline quality between CuInSe_2_ films with various photo-assistance has been investigated. In the photo-assisted electrodeposition process, the many kinds of lamps providing maximum light intensity at about 380 to 620 nm were used as light source to be irradiated onto the surface of Mo-coated soda-lime glass substrates. The results suggested effects of photo-assistance including activating surface diffusion and growing high-crystalline quality films with reduced defects during electrodeposition.

## Background

Chalcopyrite (CH) semiconductors have been applied as absorber layers for polycrystalline thin-film solar cells and extensively studied due to their importance in optoelectronic applications. CuInSe_2_ (CIS) thin films are characterized as by a suitable band gap, a high absorption coefficient that exceeds 10^5^ cm^-1^, and good stability [1]. Among ternary compounds, CIS-based thin films are the most promising for use as optical absorbers for high-efficiency solar cells as they match the solar spectrum. Various methods have been used for the growth of CIS films, such as metalorganic vapor-phase epitaxy [2], molecular beam epitaxy [3], flash evaporation [4], coevaporation [5,6], and electrodeposition [7]. Several methods based on vacuum techniques have been developed to prepare CIS layers. A photo-assisted molecular beam epitaxy (MBE) process using an Hg lamp has been investigated by Tseng et al. [8]. They found that the photo-assisted MBE process dramatically reduced the epitaxial temperature to 300°C, where photons may supply an additional energy to the film surface and activate the surface diffusion and the dissociation of Se_2_ and Se_4_ molecules [9]. Nakada et al. [10] also proposed a laser-assisted deposition (LAD) process using the MBE system. In the process, a pulsed excimer laser (*λ* =248 nm) and pulsed YAG laser (*λ* =1,064, 532, 355, and 266 nm) were irradiated onto the substrate surface during Cu (In,Ga)Se_2_ (CIGS) deposition by the three-stage process. They found the cell performance improved for all CIGS solar cells with different Ga contents, and the grain size of CIGS thin films became large and a (1 1 2)-preferred orientation was enhanced by the LAD process. The photo-assisted electrodeposition has been used to help in forming smooth and dense CIS films [10]. Accordingly, seeking possibilities for better electrodeposited film qualities, we hereby applied the photo-assistance to electrodeposition and aimed to find out the differences between electrodeposited CIS films with various wavelength photo-assistance. The crystallographic properties of CIS thin films were characterized by scanning electron microscopy (SEM), X-ray diffraction (XRD), atomic force microscopy (AFM), and Raman spectroscopy.

## Methods

**Figure 1:**
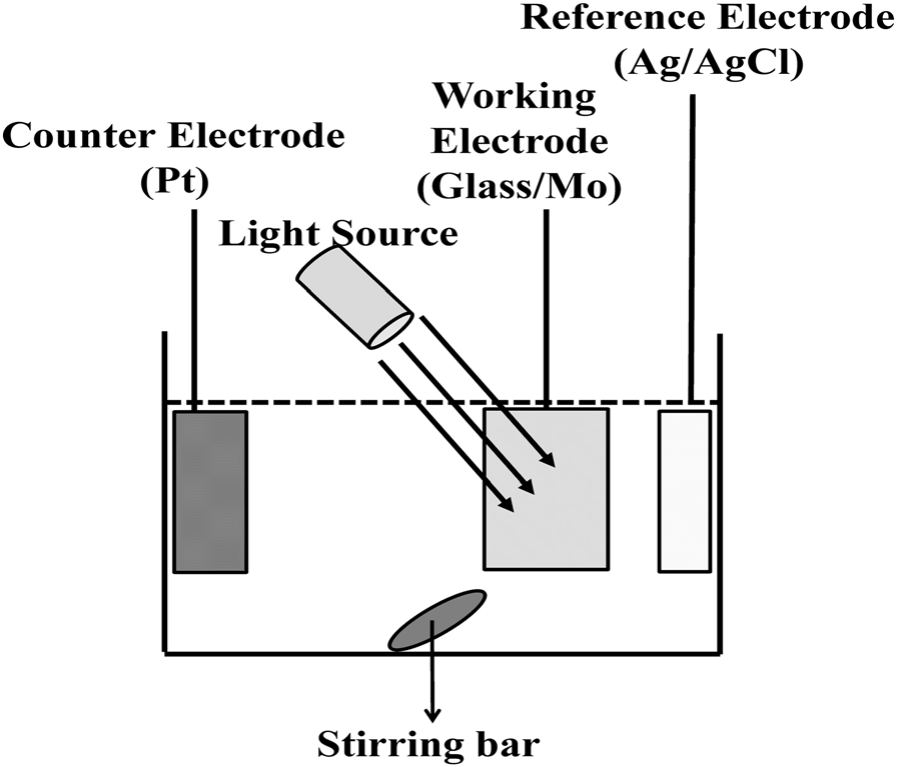
Schematic diagram of electrodeposition system.

Mo-coated soda-lime glass substrates were washed prior to CIS growth in water containing surfactant and then rinsed with deionized water. The Mo film on the glass substrate (about 1-μm thick) was deposited by radio-frequency sputtering. Codepositions of Cu, In, and Se by the electrodeposition process were performed from a bath containing 2 mM CuCl_2_, 25 mM InCl_3_, 5 mM H_2_SeO_3_, and LiCl. The pH value was adjusted to 2 using H_2_SO_4_. Figure 1 shows a schematic diagram of the electrodeposition system. The rotation speed of the magnetic stirrer was set at 50 RPM. The electrochemical measurements and electrodeposition were conducted using a conventional three-electrode potentiostat (AUTOLAB PGSTAT302 (Metrohm Autolab Inc., Utrecht, Netherlands)). A thin slice of a 99.99% pure Pt electrode with dimensions of 1 × 4 cm was employed as the counter electrode, and an Ag/AgCl electrode served as the reference electrode. Glass substrates with sputtered Mo film were used as the working electrode. The electrodeposition area was a square with dimensions of 1 × 1 cm. A magnetic stirrer was used for the stirring procedure. The rotation speed of the magnetic stirrer was set at 50 RPM. The potential was supplied by the potentiostat. The potential was fixed at -0.7 V. We applied the photo-assistance to electrodeposition and aimed to find out the differences between electrodeposited CIS films with various wavelength photo-assistance. The wavelengths of photo-assistance at about 380 to 476, 476 to 495, 495 to 570, 570 to 590, and 590 to 620 nm were applied to electrodeposition.

Growth experiments were performed in a room-temperature solution, under slow stirring of the bath. Approximately 1.7-μm-thick CIS layers were obtained after 20 min of deposition. On completion of growth, CIS films were removed from the bath, rinsed with deionized water, and dried in an argon stream.

The surface morphology and chemical composition of the films were characterized by SEM (Philips XL-40FEG (Philips, Amsterdam, The Netherlands)) and energy-dispersive spectroscopy (EDS). AFM was used in contact mode. The Raman spectra were obtained in the backscattering configuration at room temperature with unpolarized light using a spectrometer (DILOR XY 800 (J Y, Inc., Edison, NJ, USA)) and an Argon-ion laser with a 514.5-nm wavelength as the light source. The phase composition and the crystallographic structure were analyzed by XRD using a multipurpose thin-film X-ray diffractometer (Bruker D8 SSS (Bruker AXS, Inc., Madison, USA)).

## Results and discussion

**Figure 2:**
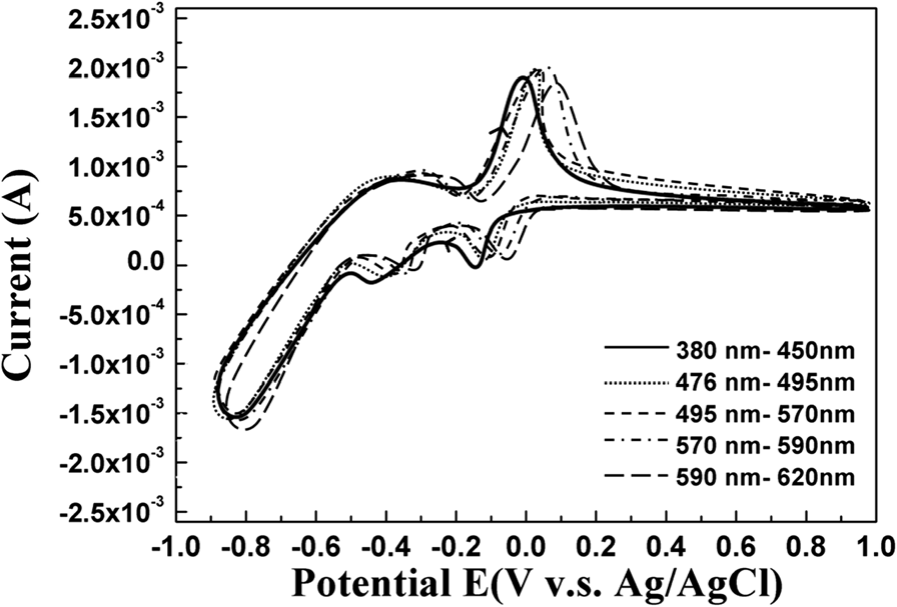
The cycle voltammogram for the oxidation potential of a Cu-In-Se solution for various wavelengths of photo-assistance.

Figure 2 shows representative linear sweep voltammogram of Cu (II), In (III), and Se (IV) ions in a hydrochloric acid, hydrogen chloride/sodium chloride, and NaCl buffer solution with distinct photo-assistance. In the beginning, a sharp and strong cathodic peak was started at -0.1 V, which is usually attributed to the formation of Cu^+^[11]:

(1) $$Cu^{2+}+e^{-}\to Cu^{+}$$

For more negative potentials, second and third peaks were observed, which must be associated with the formation of CIS. This electrode reaction has been described as a multistep process in the literature [11,12], which results in the assimilation of In^3+^ into copper selenide, leading to the formation:

(2) $$\begin{array}{l} {Cu}^{+}+Se+2e^{-}\to {Cu}_{2}Se\\ 2{\mathrm{In}}^{3+}+3H_{2}Se\;\to \;{\mathrm{In}}_{2}{Se}_{3}+6H^{+}\\ {Cu}_{2}Se+{\mathrm{In}}_{2}{Se}_{3}\to 2{\mathrm{CuInSe}}_{2} \end{array}$$

**Figure 3:**
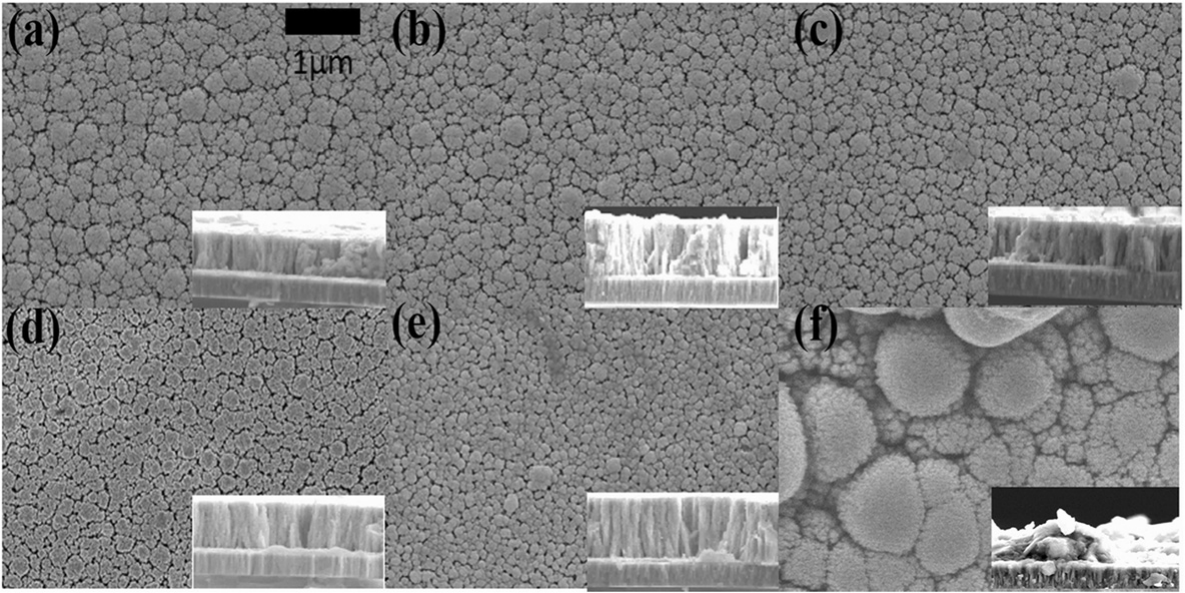
**SEM images of CIS layer deposited at various wavelengths of photo-assistance.** Wavelength at **(a)** 380 to 476 nm, **(b)** 476 to 495 nm, **(c)** 495 to 570 nm, **(d)**570 to 590 nm, **(e)** 590 to 620 nm, and **(f)** non-photo-assistance.

**Figure 4:**
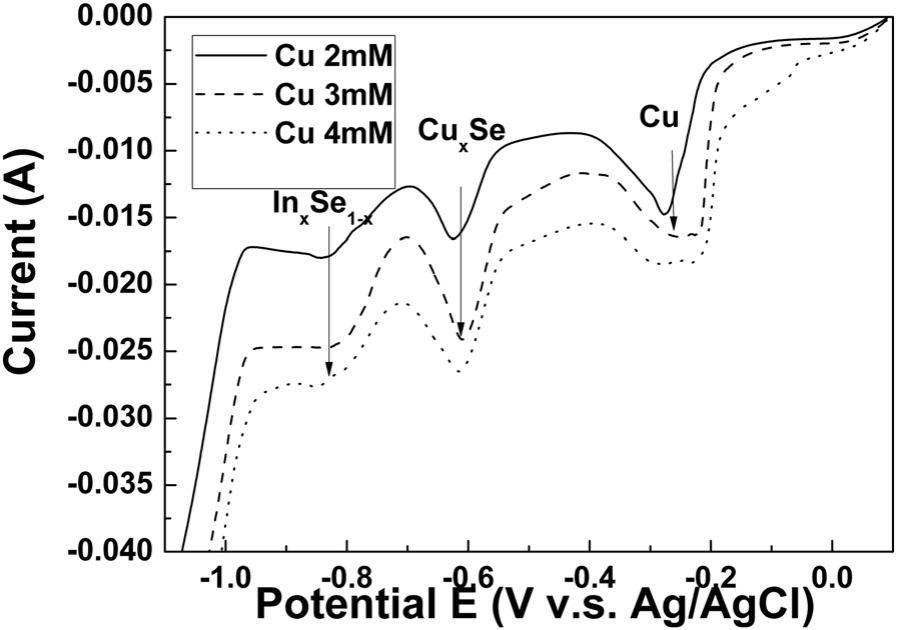
Linear sweep voltammogram for the oxidation potential of Cu-In-Se solution for various Cu concentrations.

Based on the above linear sweep voltammogram results, the intensities of peaks Cu_2_Se and In_2_Se_3_ and the current density increased with various wavelengths of photo-assistance. Moreover, the reduction potential of Cu (II), Cu_2 - x_Se and In_2_Se_3_ shifted to positive potential and prevented the evolution of H_2_ when depositing CIS on the Mo substrates. On the other hand, the limit of mass transfer rate can be raised in the various wavelengths of photo-assistance, which helps replenish ions to the substrate surface. The limit of mass transfer rate is higher than the deposition rate in the various wavelengths of photo-assistance, creating a smoother surface, as shown in Figure 3a, b, c, d, e. Figure 4 shows the linear sweep voltammogram with higher Cu concentration which helps to verify the limit of mass transport effect. The increase in current density when increasing the bath concentration indicates that the deposition reaction was originally mass transfer limited. Thus an improvement in mass transfer rate is generally beneficial for the deposition process. Table 1 shows the analysis of EDS. One can observe that the Cu/In ratio rose in the various wavelengths of photo-assistance. The analysis of EDS corresponds to the linear sweep voltammogram, where the reduction potentials of Cu (II), Cu_2 - x_Se, and In_2_Se_3_ shifted positively in the various wavelengths of photo-assistance, and it obviously shifted in the wavelength of photo-assistance at about 380 to 476 nm.

**Table 1:** EDS analysis of CIS layer deposited at various wavelengths of photo-assistance

**Various wavelengths of photo-assistance**	**Cu at %**	**In at %**	**Se at %**
Non-photo-assistance	17.88	19.26	62.86
380 to 476 nm	19.30	19.03	61.67
476 to 495 nm	21.58	18.49	59.94
495 to 570 nm	21.93	18.19	59.88
570 to 590 nm	18.11	19.33	62.56
590 to 620 nm	18.53	19.56	61.91

**Figure 5:**
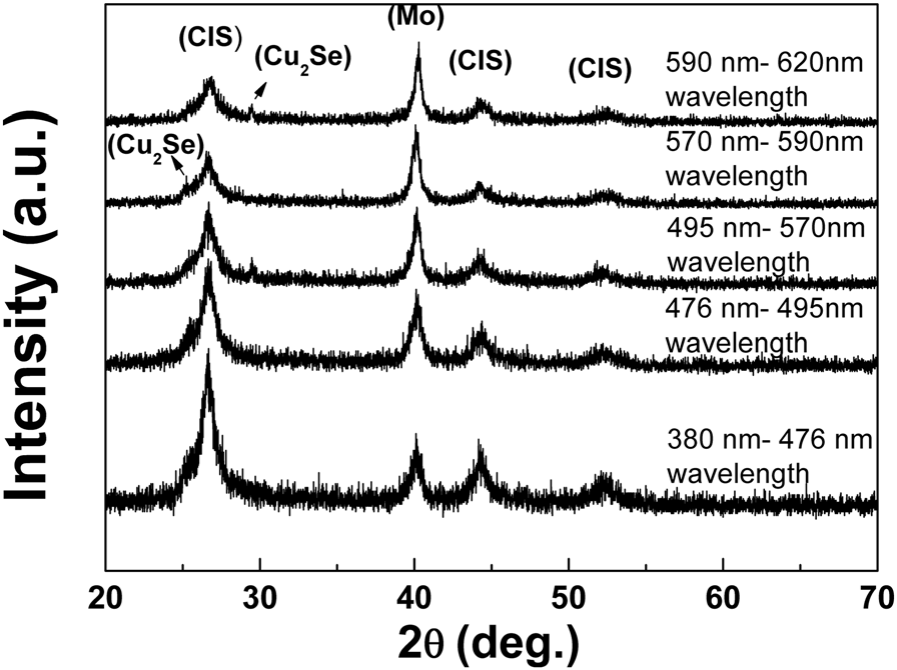
XRD patterns of as-deposited and annealed CIS films synthesized from Cu-In-Se solution at various wavelengths of photo-assistance.

The structural characterization of CIS films electrodeposited onto Mo substrates was carried out using XRD patterns within the 2θ range of 20° to 70°. XRD patterns of as-deposited CIS films in the various wavelengths of photo-assistance on Mo (1 μm)/glass substrates at -0.7 V (vs. Ag/AgCl) are presented in Figure 5. Although all peaks appear rather broad and weak which reflect a poorly crystallized material, the as-deposited films still display the three main CIS peaks; (1 1 2), (2 2 0/2 0 4), and (3 1 2/1 1 6). XRD patterns of electrodeposited CIS samples usually show peaks with broad and weak features in CIS chalcopyrite phases due to a high degree of amorphous phases and mixtures of binary and ternary compounds in the as-deposited films.

The (1 1 2) peak intensity shows more crystalline CIS with increasing intensity of photo-assistance. When the intensity of photo-assistance was increased, the CIS (1 1 2) peak intensity also increased. The (1 1 2) peak intensity shows more crystalline CIS at the wavelength of photo-assistance at about 380 to 476 nm. This is presumably because of an enhanced surface migration of reduced atoms by intensity of photo-assistance. If the atoms hold a high kinetic energy on the substrate surface, they can move to appropriate positions and form a more stable lattice plane that is a closed-packed (1 1 2) plane of chalcopyrite structure [10].

**Figure 6:**
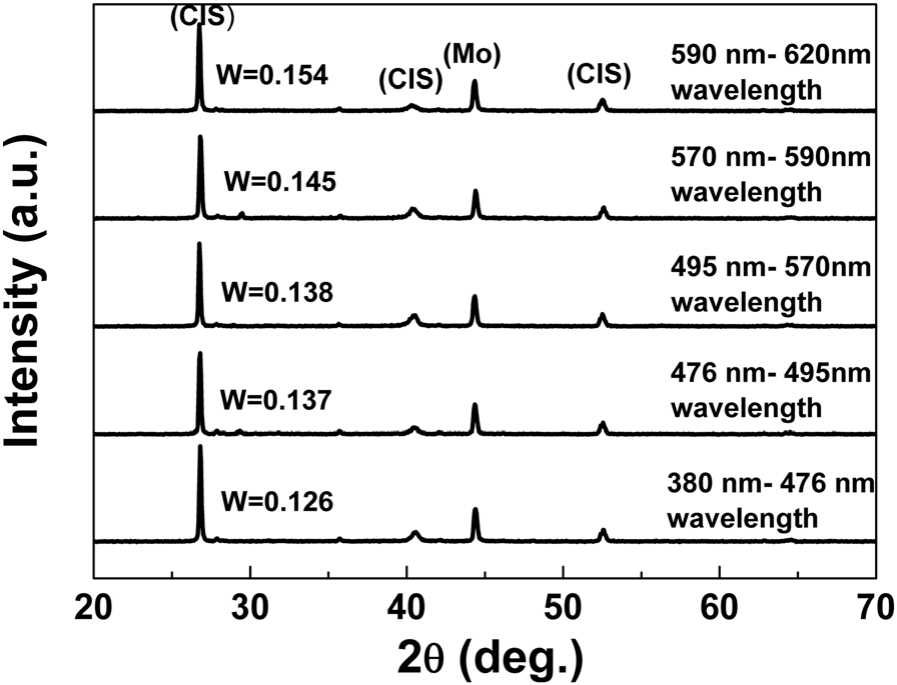
XRD patterns of annealed CIS films synthesized at various wavelengths of photo-assistance.

**Figure 7:**
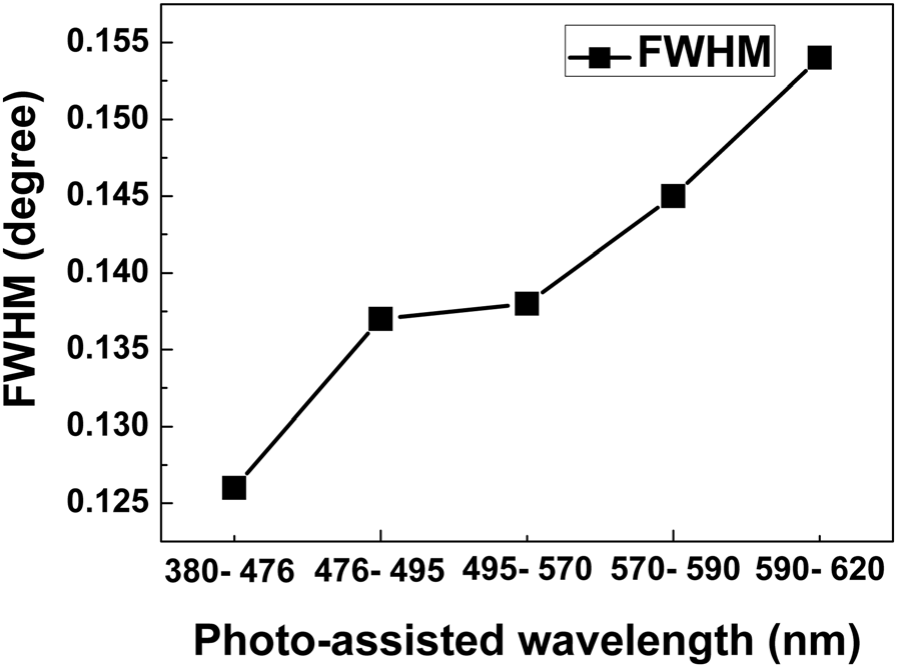
FWHM values of films synthesized from deposited CIS at various wavelengths of photo-assistance.

Figure 6 shows the results after annealing the CIS layer at 500°C for 2 min. The following annealing step generally improves crystalline properties, resulting in well-defined sharp peaks in the XRD pattern. Clear identification of the chalcopyrite crystal structure can be achieved after the annealing step in electrodeposited CIS thin films. Figure 7 shows the full width at half maximum (FWHM) in the CIS (1 1 2) peak of the films obtained at the various wavelengths of photo-assistance. Obviously, CIS films derived from photo-assistance with wavelength demonstrate lower FWHM values. This decrease in the FWHM is very important because it has been reported [12] that the efficiency of polycrystalline solar cells increases with decreasing FWHM for the absorbing materials. When the wavelengths of photo-assistance was reduced to 380 to 476 nm, the FWHM of the CIS (1 1 2) peak decreased to 0.126°. Moreover, because the wavelengths of photo-assistance were reduced, the electron voltage of the photo-assistance was raised to enhance surface migration of reduced atoms by photo-assistance [13].

**Figure 8:**
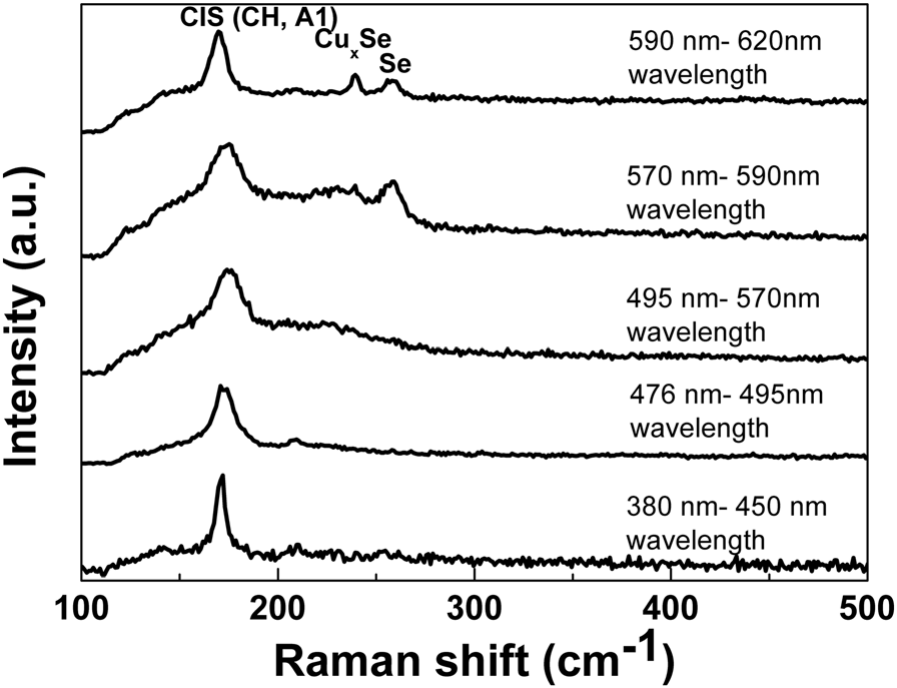
Raman spectra of CIS films obtained with various wavelengths of photo-assistance.

The annealed CIS films were also analyzed by Raman spectroscopy. Figure 8 shows the Raman spectra of annealed CIS films in various wavelengths of photo-assistance in electrodeposited CIS thin films. The main part of the spectrum is situated between 100 and 300 cm^-1^. The peak intensity of CIS CH located at A1 vibrational mode 174 cm^-1^ in the photo-assistance of near-infrared is weaker than that of CIS with the photo-assistance of near-ultraviolet. The peak intensity of CIS chalcopyrite located at E vibrational mode 216 cm^-1^ is exhibited for films in the photo-assistance of wavelengths of 380 to 476 nm and 476 to 495 nm [14]. The annealed CIS sample electrodeposited with the photo-assistance of near-infrared (570 to 590 nm and 590 to 620 nm) shows two more intense peaks; the first is situated at 240 cm^-1^ and the second at 260 cm^-1^. The peak at 240 cm^-1^ is attributed to the presence of trigonal elementary Se [15] while the peak at 260 cm^-1^ is characteristic of Cu-Se compounds [16]. There was more crystallized CIS in photo-assistance because the electron volt of photo-assistance can enhance surface migration of reduced atoms and let CIS films be evenly deposited. In addition, this CIS surface in the photo-assistance of near-ultraviolet is smoother than the CIS surface in the photo-assistance of near-infrared, because there is more surface migration of reduced atoms in the higher electron volt of photo-assistance.

## Conclusions

In summary, we have developed a method of various wavelengths photo-assistance electrodepositing for improving morphological and crystalline qualities of CIS thin films. At the photo-assistance of wavelengths of 380 to 476 nm, smooth and dense surface morphology can be achieved due to activation of surface processes with a lower potential (-0.7 V vs. Ag/AgCl). From the XRD pattern, an enhancement in (1 1 2)-preferred orientation is observed, suggesting a formation of a closed-packed (1 1 2) plane of chalcopyrite structure. Moreover, the enhancement in crystalline quality by photo-assistance of higher electron volt remains conspicuous after the following annealing step. From Raman spectroscopy, we notice a reduction in secondary phases such as Cu-Se and Se after applying photo-assistance on CIS films deposited at -0.7 V (vs. Ag/AgCl), and the increase in intensity of peaks in Raman spectra can also be referred to a better crystalline quality. In conclusion, the higher electron volt photo-assisted electrodeposition is effective to improve not only the surface morphology but crystalline quality of electrodeposited CIS thin films.

## Competing interests

The authors declare that they have no competing interests.

## Authors’ contributions

Study conception and design: T-WC, W-HL. Acquisition of data: T-WC. Analysis and interpretation of data: T-WC, Y-HS and Y-JH. Drafting of manuscript: T-WC. Critical revision: T-WC, W-HL. All authors read and approved the final manuscript.
